# Reviewer acknowledgements for 2013

**DOI:** 10.1186/1741-7015-12-9

**Published:** 2014-01-20

**Authors:** Sabina Alam

**Affiliations:** 1 BioMed Central Ltd, 236 Gray’s Inn Road, London, WC1X 8HB, UK

## Abstract

**Contributing reviewers:**

The editors of *BMC Medicine* would like to thank all our reviewers who have contributed to the journal in Volume 11 (2013).

## 

Amy Abernethy

USA

Lynne Abruzzo

USA

Adetola Adesida

Canada

Alastair Ager

USA

Paul Aisen

USA

Mohammed Ali

USA

Mouaz Al-Mallah

Saudi Arabia

Pablo Alonso-Coello

Spain

Doug Altman

UK

Danny Altmann

UK

Simone Appenzeller

Brazil

Hwyda Arafat

USA

Sevgi Aral

USA

Jari Arokoski

Finland

Richard Ashcroft

UK

Ahmad Awada

Belgium

Laurent Azoulay

Canada

Denis Azzopardi

UK

Paul Babyn

Canada

Matthias Bache

Germany

Max Oscar Bachmann

UK

Ingeborg Bajema

Netherlands

Julien Steven Baker

UK

Marian Bakermans-Kranenburg

Netherlands

Sube Banerjee

UK

Lisa Barcellos

USA

Jane Barlow

UK

Eva Bartels

Germany

Diego Bassani

Canada

Stefano Bastianello

Italy

Grant Bateman

Australia

John Batsis

USA

Manuel Battegay

Switzerland

Philip Bejon

Kenya

Tanios Bekaii-Saab

USA

Robert H. Belmaker

Israel

Derrick Bennett

UK

Sheri Berenbaum

Afghanistan

Michael Berk

Australia

David Berrigan

USA

George Bertsias

Greece

Marianne Berwick

USA

David Beverland

UK

Rakesh Bhatnagar

India

Gretchen Birbeck

Zambia

Robert Black

USA

Michael Blaha

USA

Michael Blaiss

USA

Miri Blank

Israel

Sally Blower

USA

Valentina Boeva

France

Dimitrios Bogdanos

UK

Richard Bohannon

USA

Celestino Bonura

Italy

Andre Boonstra

Netherlands

Robert Boruch

USA

John Boscardin

USA

Luigi Bouchard

Canada

Peter Bower

UK

Adrian Boyle

UK

Patricia Brennan

USA

Brian Bressler

Canada

Elisa Brietzke

Brazil

Vera Bril

Canada

Gordon Broderick

Canada

George P Browman

Canada

Martin Brüne

Germany

Traolach Brugha

UK

Susan Buchbinder

USA

Hilde Buiting

Netherlands

Luis Bujanda

Spain

William E. Bunney

USA

Alex Burdorf

Netherlands

Peter Byass

Sweden

David Cahill

UK

Gaetano Cairo

Italy

Philip Calder

UK

Giuseppe Campo

Italy

Amparo Cano

Spain

Yunxia Cao

China

Michael Carmont

UK

Rona Carroll

USA

Mauro Giovanni Carta

Italy

Philip Castle

USA

Carlo Catassi

Italy

James Cauraugh

USA

Gediminas Cepinskas

Canada

Gianfranco Cervellin

Italy

Dennis Chao

USA

Francesca Chappell

UK

Isabelle Chemin

France

Fei Chen

United States of America

Imti Choonara

UK

Gerardo Chowell

USA

George Chrousos

Greece

Kuang-Yuh Chyu

USA

James Cimino

USA

Isabel Clare

UK

Ian Clark

Australia

Myron Cohen

USA

Ian Colman

Canada

Orla Conneely

USA

Matthew Cook

Australia

Arri Coomarasamy

UK

Hoosen Coovadia

South Africa

Giampietro Corradin

Switzerland

Helena Cortez-Pinto

Portugal

Fiammetta Cosci

Italy

Nancy Cox

USA

Mary Crow

USA

Solveig Cunningham

USA

Kim Dalziel

Australia

Kaberi Dasgupta

Canada

Jennifer Davis

Canada

Daniela De Angelis

UK

Eling D. de Bruin

Switzerland

Matthijs de Hoog

Netherlands

Peter de Jonge

Netherlands

Michel de Lorgeril

France

Sandrine de Ribaupierre

Canada

Willem de Villiers

USA

Jacqueline Deen

Australia

Jessy Deshane

USA

Joseph Devaney

USA

Keertan Dheda

South Africa

Nicola Di Daniele

Italy

Mireia Diaz

Spain

Denise Doolan

Australia

Tommaso Dragani

Italy

Anne-Sophie Ducloy-Bouthors

France

Graham Dunn

UK

Juergen Eckel

Germany

Holger Eggebrecht

Germany

Jean El Cheikh

France

Adel El-Naggar

USA

Holger Eltzschig

USA

Donald Enarson

France

Meridith Englander

USA

Vikki Entwistle

UK

Dogan Erdogan

Turkey

Luis Espinoza

USA

Matthew Ewend

USA

Muller Fabbri

USA

Karl Fagerstrom

Sweden

Peter Falkai

Germany

John Fanikos

USA

Deborah Fein

USA

Abebaw Fekadu

Ethiopia

David Felson

USA

Keith Ferdinand

USA

David Fergusson

New Zealand

Mårten Fernö

Sweden

Hector Ferral

USA

Manuela Ferreira

Armenia

Edith Feskens

Netherlands

Guido Filler

Canada

Tony Fitzgerald

Ireland

Katherine Flegal

USA

Steffen Flessa

Germany

Leon Flicker

Australia

Alastair Flint

Canada

Farzin Forooghian

Canada

Francisco Forriol

Spain

Matthew Fox

USA

Laura Fratiglioni

Sweden

Kenneth Freedland

USA

Paul Frewen

Canada

Gerald Friedland

USA

Bernard F. Fuemmeler

USA

Curt Furberg

USA

Toshiaki Furukawa

Japan

Gianluca Gaidano

Italy

Frietson Galis

Netherlands

Fabrizio Gardoni

Italy

Michel Garenne

France

Paul Garner

UK

Roberto Gasparini

Italy

Steffen Gay

Switzerland

Vassilis Georgoulias

Greece

Hassan Ghomrawi

USA

Debashis Ghosh

USA

Peter Giannoudis

UK

Noel Gibney

Canada

Clare Gilbert

UK

Ruth Gilbert

UK

Alice Gilman-Sachs

USA

Gary Ginsberg

Israel

James Giordano

USA

John Gladman

UK

Julie Glanville

UK

David Glass

USA

Bertrand Godeau

France

David Goldberg

UK

Gerald Goldstein

USA

Anne Gompel

France

Assaf Gottlieb

USA

David Gough

UK

Elmar Graessel

Germany

Matthew Grainge

UK

Reuben Granich

Switzerland

William B. Grant

USA

Michael Green

USA

David Greenberg

UK

Ulla Griffiths

UK

Wolfgang Grisold

Austria

Pam Groenewald

South Africa

Alexei A. Grom

USA

Louis Grothaus

USA

Johann Gudjonsson

USA

Pal Gulbrandsen

Netherlands

Monica Guma

USA

Alistair Gunn

El Salvador

Lars L. Gustafsson

Sweden

Balazs Györffy

Hungary

Mark Haacke

USA

Kare Birger Hagen

Norway

M Elizabeth Halloran

USA

Dennis Han

USA

Jing-Dong Han

China

Johnni Hansen

Denmark

Rebecca Hardy

UK

Robin Haring

Germany

Kathleen Harrington

USA

Ross Harris

UK

Jan Gunnar Hatlebakk

Norway

Pierre Hausfater

France

Robert Hawkins

USA

Gregory Heath

USA

Jerry Heindel

USA

Rob Herbert

Australia

Sabine Herpertz

Germany

Tiberiu Hershcovici

USA

Andrew Hill

New Zealand

Caroline Hing

UK

Ze'ev Hochberg

Israel

Allison Hodge

Australia

Michael Holick

USA

Bruce Hollis

USA

Geoffrey Holmes

UK

Masaru Horio

Japan

Chi-yuan Hsu

USA

Han Hwa Hu

Taiwan

Johnny Huard

USA

Carroll W.Hughes

USA

Berthold Huppertz

Austria

Sara Hurvitz

USA

Henrik Husted

Denmark

Dietmar Werner Hutmacher

Australia

Steve Iliffe

UK

Rob Imrie

UK

Giuseppe Ippolito

Italy

Nobukazu Ishizaka

Japan

Felice Jacka

Australia

Debra Jackson

South Africa

Kitty Jager

Netherlands

Jeroen Paul Jansen

USA

David Jenkins

Canada

Gerald Jerome

USA

Derek Jewell

UK

Masamine Jimba

Japan

Félix Javier Jiménez-Jiménez

Spain

Anne Joerns

Germany

Russell Joffe

USA

Tom Johnston

Canada

Jussi Jokinen

Sweden

Hayley Jones

UK

Håkan Jonsson

Sweden

Tanja Jovanovic

USA

Nicole Juffermans

Netherlands

Steven Julious

UK

Stevo Julius

USA

Risto Kaaja

Finland

Philipp Kahlert

Germany

James G. Kahn

USA

Joachim Kalden

Germany

Ashish Kamat

USA

Katherine Kaplan

USA

S. Ananth Karumanchi

USA

Jeffrey Katz

USA

Heath Kelly

Australia

Cyril Kendall

Canada

Martin Kennedy

New Zealand

Rose Anne Kenny

Ireland

Lars Kessing

Denmark

Monique Kilkenny

Australia

Hunkyung Kim

Japan

Gary King

USA

Jun-ichi Kira

Japan

Irving Kirsch

UK

Jorg Kleeff

Germany

Jos Kleijnen

UK

Patrick Kolsteren

Belgium

Amos Korczyn

Israel

Eline Korenromp

Switzerland

Shyam Kottilil

USA

Aleksander Krag

Denmark

Saurabh Kumar

Australia

Antonio La Cava

USA

Pirjo Laakkonen

Finland

Daniel Labovitz

USA

Massimo Labra

Italy

Bonnie LaFleur

USA

Rikard Landberg

Sweden

Hans Lassmann

Austria

Helen Lavretsky

USA

Harold Lebovitz

USA

Peter Lee

UK

Youngjo Lee

South Korea

Sam Leinster

UK

Michael Leiter

Canada

Sigurd Lenzen

Germany

Anne Lethaby

New Zealand

Michael Lever

New Zealand

Francesca Levi-Schaffer

Israel

Juan C. Leza

Spain

Chin-Shang Li

USA

Jairam Lingappa

USA

Francesca Little

South Africa

Li Liu

USA

Antonio Llombart-Bosch

Spain

Fiona Lobban

UK

Yoon Kong Loke

UK

Anna Lokshin

USA

Carl Lombard

South Africa

Alan Lopez

Australia

Christopher Lowry

USA

Jayne Lucke

Australia

Jens Lundgren

Denmark

Knut E.A. Lundin

Norway

Paul Lysaker

USA

Michael Maes

Thailand

Antoine Magnan

France

Sabatino Maione

Italy

Kathryn Maitland

UK

Milos Maksimovic

Serbia

Giovanni Malferrari

Italy

Rayaz Malik

UK

Jeffrey Marks

USA

Stefan Marlovits

Austria

Maurice Mars

South Africa

Miguel Martinez-Gonzalez

Spain

Luca Mascitelli

Italy

Gianluca Masi

Italy

Colin Mathers

Switzerland

Ilan Matok

Israel

Cyril Mauffrey

USA

Clio Mavragani

Greece

Colleen Maxwell

Canada

Guillermo Mazzolini

Argentina

Linda McCaig

USA

Sheena McCormack

UK

Karen McKenzie

UK

Chris McManus

UK

Robert McMurray

USA

Iain McNeish

UK

Lisa McShane

USA

Pascal Meier

UK

Onno Meijer

Netherlands

Susan Meikle

USA

Giampaolo Merlini

Italy

Pier Luigi Meroni

Italy

Lucio Miele

USA

Gregoire Millet

Switzerland

Edward Mills

Canada

Anoop Misra

India

Geoffrey Mitchell

Australia

Atsushi Mizoguchi

USA

Martin Möckel

Germany

Malcolm Edward Molyneux

Malawi

Manuel Monreal

Spain

Michael A. Mont

USA

Scott Montgomery

Sweden

Timothy Mosher

USA

Haralampos Moutsopoulos

Greece

Sandy Munger

USA

M. Hassan Murad

USA

John Myburgh

Australia

Dean Myers

USA

Jeffrey Myers

USA

Frederick Naftolin

USA

Andrew Naidech

USA

Bijan Najafi

USA

Brahmajee Nallamothu

USA

Luis Nannini

Argentina

Denis Nash

USA

Urs Nater

Germany

Thumbi Ndung'u

South Africa

Dale Needham

USA

Charles Nemeroff

USA

John Nemunaitis

USA

Britt-Ingjerd Nesheim

Norway

Randolph Nesse

USA

Frank Neuner

Germany

Antonino Nicoletti

France

Dorte Lisbet Nielsen

Denmark

Hiroshi Nishiura

Japan

Enzo Nisoli

Italy

Rachel Norman

UK

Susan L. Norris

USA

John Nurnberger

USA

Eric Nylen

USA

Rima Obeid

Germany

Tim Oberlander

Canada

Masaki Ohsawa

Japan

Tim Olds

Australia

Altan Onat

Turkey

Bregje D. Onwuteaka-Philipsen

Netherlands

Jan Erik Otterstad

Norway

Vural Ozdemir

Canada

Andrea Pace

Italy

Clive Page

UK

Nam-Jong Paik

South Korea

Nikolaos Papanas

Greece

Evridiki Papastavrou

Cyprus

Kosmas Paraskevas

Greece

Jiri Parenica

Czech Republic

Carmine Pariante

UK

David Parkinson

USA

Terence Partridge

USA

Freidemann Paul

Germany

Paul Pavlidis

Canada

Ed Peile

UK

Kenneth Perkins

USA

Vanamail Perumal

India

Donald Phinney

USA

Susan Picavet

Netherlands

Robert Pickard

UK

Jan Piek

Netherlands

Munir Pirmohamed

UK

Lionel Piroth

France

Paola Pisani

Italy

David Plante

USA

William Powderly

USA

Nathan Price

USA

Patricia Price

Australia

Darwin Prockop

USA

Leandro Provinciali

Italy

Milo Puhan

USA

Vinod Pullarkat

USA

Francesco Puppo

Italy

Paolo Puppo

Italy

Chengxuan Qiu

Sweden

Houshang Rafatpanah

Iran

Anisur Rahman

UK

Kilian Rapp

Germany

Stephen Rathbun

USA

Caroline Relton

UK

Jan Remme

France

Andrew Renehan

UK

Stephen Resch

USA

Richard Rheingans

USA

José Ribeiro

Portugal

Todd Richards

USA

Thomas Richie

USA

Hans Rieder

Switzerland

Michael Rieder

Canada

Maria Luiza Riesco

Brazil

Shawn Ritchie

Canada

Emanuel Rivers

USA

Trudie Roberts

UK

Nicolas Roche

France

Graeme Rocker

Canada

Sabine Rohrmann

Switzerland

Noel Rose

USA

Joseph Ross

USA

Jacques Rossouw

USA

Carlo Maria Rotella

Italy

Michael Roth

Switzerland

Thomas Roth

USA

Jean Roudier

France

Craig Rubens

USA

Reiner Rugulies

Denmark

Monika Safford

USA

Chris Salisbury

UK

Vaishali Sanchorawala

USA

Ferran Sanz

Spain

Leslie Satin

USA

Elke Schaeffner

Germany

Wolfgang Schaper

Germany

Werner Scheithauer

Austria

Maren Scheuner

USA

Neil Schluger

UK

Michael Schull

Canada

Carolyn Schwartz

USA

David Scott

USA

Stephen Scott

UK

Caroline Sewry

UK

Daniel Sexton

USA

Haleema Shakur

UK

Basil Sharrack

UK

Christopher Shaw

Canada

Imad Sheiban

Italy

Marv Shepherd

USA

William Sheremata

USA

Kenji Shibuya

Japan

Hai-Rim Shin

Philippines

Sonya Shin

USA

Yehuda Shoenfeld

Israel

Nikos Siafakas

Greece

Rosa Sicari

Italy

Yardena Siegman-Igra

Israel

Lori Silveira

USA

Robert Silver

USA

Marian Simka

Poland

Nora Singer

USA

Dario Siniscalco

Italy

Salvatore Siracusano

Italy

Freddy Sitas

Australia

Jens Siveke

Germany

Jingyuan Song

China

Kirk Spencer

USA

Rakesh Srivastava

USA

Stephen Stansfeld

UK

Carsten Stephan

Germany

Sarah Stevens

UK

Sarah Stewart-Brown

UK

Rob Stockley

UK

Mark Stoeckle

USA

Henrik Stovring

Denmark

Jørund Straand

Norway

Babill Stray-Pedersen

Norway

Lillian Sung

Canada

Dipika Sur

India

Ian Swain

UK

Artur Swiergiel

Poland

Nicola Tambasco

Italy

Donald Tashkin

USA

Nicholas Taylor

Australia

Richard Taylor

Australia

Cenk Tek

USA

Olle Ten Cate

Netherlands

Mallika Tewari

India

Kim Thomas

UK

Wayne Thomas

Australia

Boyd Taylor Thompson

USA

John Thompson

UK

James Timmons

UK

Giuseppe Toffoli

Italy

Robertus Tollenaar

Netherlands

Richard Troiano

USA

Tony Tse

USA

Georgios Tsivgoulis

Greece

R. Michael Tuttle

USA

Andre Tylee

UK

Pippa Tyrrell

UK

Obioha Ukoumunne

UK

Nigel Unwin

Barbados

Mukund Uplekar

Switzerland

Cara Usher

Ireland

Claire Vale

UK

Louis Valiquette

Canada

Françoise van Bambeke

Belgium

Hugo van Bever

Singapore

Peter van de Kerkhof

Netherlands

Robert-Jan van Geuns

Netherlands

Linda van Horn

USA

Niels van Royen

Netherlands

Marco Aurelio Vaz

Brazil

Stefano Vella

Italy

Jos Verbeek

Finland

Bruno Vincenzi

Italy

Arnauld Villers

France

Stein Emil Vollset

Norway

Umberto Volta

Italy

Frederick Vomsaal

USA

Theo Vos

USA

Hubertus Vrijhoef

Singapore

Dennis Wall

USA

James Walsh

USA

Tony Waterston

UK

Stuart Watson

UK

Jadwiga Wedzicha

UK

Clement M.L. Werner

Switzerland

Colin West

USA

Gunilla Westergren-Thorsson

Sweden

Robyn Whittaker

New Zealand

Tim Wilkinson

New Zealand

Alun Williams

UK

Brian Williams

South Africa

Glenn Williams

USA

Jack Williams

Canada

Charles Wiysonge

South Africa

Peter Wolf

Denmark

Charles Wood

USA

Angela Woodiwiss

South Africa

Mark Woodward

Australia

Hermann Wrigge

Germany

Hidenori Yamasue

Japan

David Yorston

UK

Bessie Young

USA

Bryan Young

Canada

Greg Zaharchuk

USA

Michele Zappella

Italy

Nathan Zasler

USA

Jennifer Zeitlin

France

George Zhang

USA

Qin Zhang

USA

Zhengdong Zhang

China

Yinshan Zhao

Canada

Frans Zitman

Netherlands

Armin Zittermann

Germany

Wainer Zoli

Italy

Merrick Zwarenstein

Canada

Matjaz Zwitter

Slovenia

## Pre-publication history

The pre-publication history for this paper can be accessed here:

http://www.biomedcentral.com/1741-7015/12/9/prepub

